# Influence of Electrode–Tissue Contact Area on Parameter Sensitivity in Electrosurgical Monopolar Soft Coagulation: A Multiphysics Finite Element Study

**DOI:** 10.3390/s26061975

**Published:** 2026-03-21

**Authors:** Christoph Busch, Stefan J. Rupitsch, Knut Moeller

**Affiliations:** 1 Institute of Technical Medicine (ITeM), Furtwangen University, Jakob-Kienzle-Str. 17, 78054 Villingen-Schwenningen, Germany; christoph.busch@hs-furtwangen.de; 2 Department of Microsystems Engineering, Faculty of Engineering, University of Freiburg, 79110 Freiburg, Germany; stefan.rupitsch@imtek.uni-freiburg.de

**Keywords:** electrosurgical soft coagulation, electrode–tissue contact area, parameter sensitivity analysis, finite element modeling, multiphysics simulation

## Abstract

Physics-based simulations are increasingly used to improve understanding of electrosurgical processes and to enable model-based estimation of tissue state when direct sensing is limited. The performance of such simulation-based virtual sensing approaches strongly depends on an accurate representation of the electrode–tissue interface. Despite its central role in electrical and thermal coupling, the influence of the electrode–tissue contact area has received limited attention in existing simulation studies. In this work, the influence of the electrode–tissue contact area on the sensitivity of key temperature-dependent tissue parameters was investigated for electrosurgical monopolar soft coagulation. Using a multiphysics finite element model under controlled boundary conditions, the sensitivity of maximum temperature development and necrotic tissue volume formation was analyzed with respect to varying contact areas and initial values of electrical conductivity, thermal conductivity, and effective heat capacity. The results demonstrate that parameter sensitivities are strongly contact-area-dependent. Electrical conductivity exhibits the most pronounced influence, particularly at larger contact areas, while thermal conductivity remains of minor relevance. In contrast, effective heat capacity significantly affects necrotic tissue volume formation, with increasing sensitivity for larger contact areas. These findings emphasize the importance of accurately accounting for electrode–tissue contact conditions in simulation-based analyses and clarify how contact-area-dependent sensitivities influence model-based tissue state estimation in electrosurgical coagulation.

## 1. Introduction

The surgical use of high-frequency (HF) alternating current (AC) to exploit thermal effects for therapeutic purposes is a well-established practice and is routinely employed in operating rooms worldwide. Nevertheless, complications such as unforeseen and unintended tissue burns [1,2,3,4,5], as well as thermal irritation or even damage to nerves [6,7,8,9], continue to pose significant clinical challenges. Some of these complications are closely related to the specific surgical technique used and the anatomical location of the procedure. Notably, fewer complications are reported in bipolar applications compared to monopolar procedures [4,10], primarily because the current in bipolar configurations is confined to a localized tissue region, thereby minimizing thermal damage to adjacent structures. In contrast, monopolar applications involve current flow through a considerably larger portion of the patient’s body, which inherently increases the risk of unintended injury [2,3,4,9]. Despite this, the versatility and flexibility of monopolar techniques ensure their continued widespread use in surgical practice.

The development of a control application capable of characterizing biological tissue in real time prior to energy delivery, thereby enabling tissue-specific dosimetry with the minimum necessary electrical power, represents a long-term technological objective and appears particularly challenging in monopolar applications. This complexity arises due to the presence of multiple types of tissue, including their boundary surfaces, existing between the active and the neutral electrode. Furthermore, the user handling leads to various contact areas of the electrode–tissue interface and thus directly affects the impedance-based electrical feedback and adds further potential influencing factors. However, such control concepts, which aim to estimate tissue state based on prior knowledge in conjunction with electrical and thermal signals, are generally considered promising approaches to enhance procedural safety, particularly in robotic applications [9], and complement existing control systems [11]. In addition to a sufficiently validated database of diverse tissue types and their characteristic properties, it is essential to identify which tissue parameters are most relevant to achieve the desired therapeutic effect. Instead of reproducing clinical variability directly, such understanding necessitates controlled investigations of underlying electro-thermal mechanisms governing parameter influence.

This fundamental understanding is challenging to obtain experimentally because of the complexity of the underlying physical processes and the substantial effort required for reliable in vivo or ex vivo measurement. In this context, finite element modeling (FEM) provides a powerful tool for investigating complex multiphysics phenomena. It facilitates, inter alia, the targeted identification of parameters that significantly influence the therapeutic effect, thereby offering important insights into the mechanisms governing electrosurgical procedures. Such analyses have already been conducted for several of these applications, including radiofrequency (RF) ablation, microwave ablation, and open surgical monopolar applications. For instance, Hall et al. [12] and Wang et al. [13] investigated model parameters for RF ablation over durations exceeding one minute and concluded that the electrical conductivity of the tissue is a major determinant of the thermal damage. Hall et al. [12] further noted that thermal conductivity becomes relevant primarily during the cooling phase of the procedure. In the context of microwave ablation, Sebek et al. [14] emphasized the importance of the electrical conductivity over a ten-minute application period, whereas heat capacity was found to be negligible.

While general sensitivity patterns of relevant parameters may be observed across different studies, some findings diverge. For instance, Karaki et al. [15] reported that both electrical conductivity and heat capacity significantly influenced the simulated temperature outcome in their study on an open electrosurgical monopolar application. These discrepancies highlight that the impact of individual parameters strongly depends on the underlying physical assumptions, boundary conditions, and the manner in which the parameters are implemented in the model. This is particularly true for thermally induced processes, such as Joule heating in biological tissues, where temperature-dependent behavior plays an important role.

In this study, we focus on the application of electrosurgical monopolar soft coagulation. This technique involves the delivery of a sinusoidal voltage signal at a frequency of 350 kHz via an active electrode. In the present scenario, an open surgical application with a ball electrode with a diameter of 4 mm is employed as the active electrode. The applied HF voltage induces resistive heating at the electrode–tissue interface, resulting in a localized increase in tissue temperature. When the tissue reaches the temperature range corresponding to the boiling point of water, intracellular and extracellular fluids begin to vaporize. This vaporization leads to local tissue contraction, which contributes to hemostasis by sealing small blood vessels. Depending on the temperature achieved and the duration of thermal exposure, irreversible damage to cellular structures occurs. This thermal injury ultimately leads to the formation of necrotic tissue in the affected region.

Typically, this application is used in surgery to achieve targeted and localized thermal tissue destruction for hemostasis. The duration of the application process varies from milliseconds to a few seconds, and is generally sufficient to achieve effective hemostasis. Considering the heat transfer in the tissue on a macroscopic level and the relatively slow heating of the tissue (milliseconds to seconds), particularly in the case of electrosurgical soft coagulation, this application is suitable for an investigation based on heat transfer laws.

In one of our previous investigations on electrosurgical monopolar soft coagulation, we analyzed the effect of varying electrode–tissue contact areas on the heating process and subsequent formation of thermal necrosis [16]. We demonstrated that the contact area exerts a significant influence on Joule heating and the resulting necrotic tissue volume. As discussed there, the contact area at the application side has received limited attention in prior analyses, including those involving sensitivity studies by other research groups. Therefore, in the present study, our aim is to address this gap by systematically investigating how variations in electrode–tissue contact area modulate the sensitivity of temperature-dependent tissue parameters within a representative physiological parameter space. Consequently, the objective of this research is to analyze the question: How does the electrode–tissue contact area influence the relative sensitivity of temperature-dependent tissue parameters governing maximum temperature development and thermal tissue necrosis formation during electrosurgical monopolar soft coagulation?

Our objective is thereby to provide deeper insight into the causal mechanism of the combined impact of how electrode–tissue contact conditions and temperature-dependent tissue parameters jointly influence simulated thermal behavior. This requires the isolation of fundamental electro-thermal parameter sensitivity within a controlled modeling environment. Therefore, the present study investigates quasi-stationary electrode–tissue contact conditions under controlled boundary assumptions. The objective is not to reproduce complete clinical procedures, but to analyze how defined interface states influence parameter dominance within a coupled multiphysical framework.

The present investigation is conducted exclusively using a simulation-based approach. This approach is grounded in well-established physical laws and informed by findings of other research groups. In the following sections, we first provide a concise overview of the methodology employed and the fundamental physical principles underlying the model. Subsequently, the finite element simulation model and the selected tissue parameters used in this study are introduced. The simulation results are then presented and analyzed. The discussion begins with a sensitivity analysis of three temperature-dependent tissue parameters relevant to the investigated application, using the developed model. In a second step, we explore how this sensitivity is affected by variations in the electrode–tissue contact area. Finally, the results are summarized and classified.

## 2. Materials and Methods

Based on established mathematical formulations and fundamental physical laws, a multiphysics simulation model was previously developed in an earlier study [16]. To address the current research objective and answer the corresponding scientific question, the FEM was applied to this model. The simulation framework comprises a mechanical, thermal, and electrically coupled multiphysics model designed to replicate the process of electrosurgical soft tissue coagulation. The model integrates structural mechanical properties, such as the hyperelastic behavior of biological soft tissue, with thermal and electrical characteristics to simulate Joule heating induced by the application of an alternating HF current in a simplified manner. In addition to these fundamental physical phenomena, the model also incorporates the thermally induced cell death process, allowing estimation of necrotic tissue formation.

In the following section, the governing physical equations as well as the simulation setup, including boundary and initial conditions, are briefly outlined. For a comprehensive description of the underlying model, the reader is referred to our previous publication, see [16]. The simulations were conducted using COMSOL Multiphysics^®^ version 6.3 [17] on a high-performance workstation equipped with an AMD Ryzen Threadripper PRO 5995WX processor (Santa Clara, CA, USA) and 512 GB of RAM. For post-processing and graphical visualization, the simulation data were exported from COMSOL Multiphysics^®^ and subsequently imported into MATLAB R2025b (MathWorks, Natick, MA, USA) [18], where further analysis and data visualization were performed.

### 2.1. Finite Element Model & Governing Equations

In modeling the electrosurgical soft coagulation process of biological soft tissue, it is essential to accurately represent both the mechanical and thermo-electric properties of the tissue. At the onset of the application, the coagulation electrode comes into contact with the soft tissue. Depending on the pressure exerted by the electrode and the mechanical characteristics of the tissue, a contact area is formed at the interface between the two, resulting from deformation of the soft tissue. Assuming that this contact area remains constant throughout the application, an electric voltage is applied. As a result of various influencing factors such as the contact area, the applied voltage, and the intrinsic properties of the underlying tissue, Joule heating predominantly occurs within the soft tissue in the immediate vicinity of the contact area. The resulting temperature rise and its duration lead to thermal cell damage. Therefore, to accurately simulate the soft coagulation process in a numerical model, both the equation of motion and the heat conduction equation must be considered.

The soft tissue was assumed to be isotropic, incompressible, and hyperelastic, and its mechanical behavior was modeled using a Neo-Hookean material formulation [19,20,21,22]. The coupling between the equation of motion and the hyperelastic behavior of the tissue is expressed through the second Piola-Kirchhoff stress tensor. The spatial and temporal distribution of heat is described by the partial differential equation (PDE) that governs heat conduction. An additional source term in the heat equation accounts for the externally supplied electrical energy, which results from the applied electrical voltage. Assuming negligible displacement currents due to the HF alternating voltage of 350 kHz typically used in the soft coagulation application, a quasi-electrostatic approach is adopted [23]. As a result, the electric potential fields within the materials can be described by the Laplace equation. Applying Ohm’s law in combination with the field-potential relationship, which defines the connection between the electric field and the electric potential, yields the following coupled heat equation:

(1) $$\rho C_{\mathrm{eff}}\left(T\right)\frac{\partial T}{\partial t}=\nabla \cdot \left(k\left(T\right)\nabla T\right)+\sigma \left(T\right){\left|\nabla V_{\mathrm{rms}}\right|}^{2},$$

where $C_{\mathrm{eff}}\left(T\right)$, k(T), and σ(T) are temperature-dependent effective heat capacity, thermal conductivity, and electrical conductivity, respectively. The variable *T* denotes the temperature at a given time *t*, under an applied root-mean-square (RMS) voltage $V_{\mathrm{rms}}$ and material density ρ. The potential $V_{\mathrm{rms}}$, representing the RMS value of a typical HF voltage used in soft coagulation, is set to 98V.

The coupling of mechanical, thermal, and electrical interactions at the interface $\Gamma_{\mathrm{et}}$ between the electrode and the tissue was implemented using the following boundary conditions:

(2) $$\begin{array}{cc} n\cdot q_{\mathrm{et}}= & -k\left(T\right)\frac{\partial T}{\partial n}=\frac{T_{e}-T_{t}}{R_{\mathrm{eq}}} \end{array}$$

(3) $$\begin{array}{cc} n\cdot J_{\mathrm{et}}= & -h_{c}\left(V_{e}-V_{t}\right) \end{array}$$

The scalar product of the surface normal vector n and the heat flux vector $q_{\mathrm{et}}$ in (2) represents the heat flux component directed across the interface from the electrode into the tissue. Here, $T_{e}$ and $T_{t}$ denote the temperatures of the electrode and the tissue, respectively. For the thermal contact resistance $R_{\mathrm{eq}}$ at the interface, a constant value of $100\;\mu {\mathrm{Km}}^{2}/W$ was assumed [24]. Similarly, the scalar product of the surface normal vector n and the current density vector $J_{\mathrm{et}}$ in (3) describes the electrical interaction at the interface between the electrode and the tissue. The term $h_{c}$ denotes the contact conductance of the interface, while $V_{e}$ and $V_{t}$ represent the electrical potentials at the electrode and tissue, respectively.

The electrode–tissue interface is modeled using distinct electrical and thermal contact formulations. The electrical contact conductance is calculated using the Mikic elastic correlation and is dependent on the contact pressure and the effective electrical conductivity of the materials in contact. Given the temperature-dependent nature of tissue conductivity, the electrical contact conductance evolves during the simulation and contributes to the electro-thermal feedback mechanism. In contrast, the thermal contact resistance is maintained at a constant level. In the soft coagulation investigation here, heat transfer is dominated by volumetric conduction within the tissue rather than by interface limitation. This makes thermal contact resistance a secondary effect compared to electrical current redistribution. A detailed derivation of the contact model can be found in the work of Busch et al. [16].

Electromagnetic losses due to Joule heating in the tissue at the electrode–tissue interface lead to localized temperature increases, which in turn alter the thermophysical properties of biological tissue [25,26,27]. In addition to this temperature-dependent behavior, phase change phenomena (particularly the evaporation of tissue water occurring at approximately 100 °C) substantially influence the heating dynamics during electro-thermal coagulation [23]. These effects were incorporated into the model through temperature-dependent formulations of the most relevant material parameters. The electrical conductivity σ(T) is defined as:

(4) $$\begin{array}{c} \sigma \left(T\right)=\left\{\begin{array}{cc} \sigma_{\mathrm{ref}}\left[1+0.02(T-T_{\mathrm{ref}})\right]\cdot W\left(T\right) & T<100\;{}^{\circ}C\\ 0.01\;S/m & T\geq 100\;{}^{\circ}C \end{array}\right., \end{array}$$

where $\sigma_{\mathrm{ref}}$ is the electrical conductivity determined at the reference temperature $T_{\mathrm{ref}}=25\;{}^{\circ}C$, and W(T) is a logistic function representing the tissue water content. Water evaporation begins around 80 °C, and the full functional form of W(T) is given in (7).

The thermal conductivity k(T) is defined as a linear function of temperature:

(5) $$k\left(T\right)=k_{\mathrm{ref}}+k_{1}\left(T-T_{\mathrm{ref}}\right),$$

where $k_{\mathrm{ref}}$ denotes the thermal conductivity determined at $T_{\mathrm{ref}}=25\;{}^{\circ}C$, and $k_{1}=1.161\;\mathrm{mW}/\left(m\cdot K\cdot {}^{\circ}C\right)$ controls the rate of change with temperature [26].

To account for the influence of tissue water evaporation on heat storage, the effective heat capacity $C_{\mathrm{eff}}\left(T\right)$ is defined following the approach of Yang et al. [28] and Chen et al. [29]. This formulation combines the heat capacity contributions from the dry tissue $C_{t}$, tissue water $C_{w}$, and the latent heat of water evaporation $C_{l}$:

(6) $$C_{\mathrm{eff}}\left(T\right)=\underset{C_{t}}{\underset{︸}{C_{\mathrm{ref}}\left(1-W_{\mathrm{ref}}\right)}}+\underset{C_{w}}{\underset{︸}{C_{\mathrm{ref}}W\left(T\right)}}+\underset{C_{l}}{\underset{︸}{\frac{L\rho_{H_{2}O}}{\rho }\frac{\partial W(T)}{\partial T}}},$$

where $C_{\mathrm{ref}}$ is the tissue specific heat capacity at 37 °C, $W_{\mathrm{ref}}=0.70$ is the initial tissue water content [30], L=2260kJ/kg is the latent heat of water, and $\rho_{H_{2}O}=1000\;\;\mathrm{kg}/m^{3}$ is the density of water.

The temperature-dependent water content is modeled using a logistic loss function:

(7) $$W\left(T\right)=\frac{W_{\mathrm{ref}}}{1+\exp(0.35\left(T-T_{s}\right))}+W_{\mathrm{end}},$$

where $T_{s}=100\;{}^{\circ}C$ is the boiling point of water, and $W_{\mathrm{end}}=0.001$ represents the residual water content after complete evaporation [16].

**Figure 1:**
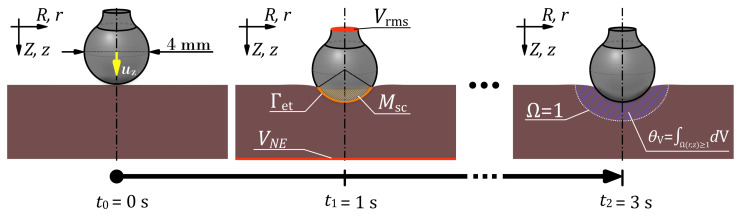
Schematic representation of the simulation procedure at three distinct time points: $t_{0}$, $t_{1}$, and $t_{2}$. At the initial time point, $t_{0}$, the diameter of the ball electrode is shown along with the z-component of the displacement vector, u. At the time point $t_{1}$, the boundary potentials $V_{\mathrm{rms}}$ and $V_{\mathrm{NE}}$ are indicated, representing the area where the root-mean-square (RMS) voltage of 98V and the neutral-electrodes potential of 0V are applied, respectively. Additionally, the formed contact area $M_{\mathrm{cs}}$ together with the interface $\Gamma_{\mathrm{et}}$, where the heat transfer and electrical contact between the electrode and the tissue is considered, are indicated. At the final time point, $t_{2}$, the boundary of the necrotic tissue region is illustrated, corresponding to a damage parameter value of Ω=1. Furthermore, the blue shaded area symbolizes the necrotic tissue volume, $\theta_{V}$, and its definition.

The thermally induced tissue necrosis was quantified using a first-order Arrhenius-type equation. In this framework, the damage process is modeled as a cumulative reaction over time, allowing both the magnitude and the duration of the temperature exposure to be taken into account [31,32]. The dimensionless damage parameter Ω(τ) represents the extent of tissue injury at time τ and is obtained by integrating the temperature-dependent damage rate over time. The corresponding relation is given by:

(8) $$\Omega \left(\tau \right)=\int_{0}^{\tau }A_{0}\cdot \exp\left(\frac{-E_{a}}{R\cdot T(t)}\right)dt,$$

where *R* denotes the universal gas constant, while $E_{a}$ and $A_{0}$ are tissue-specific parameters representing the activation energy and frequency factor, respectively, for the thermal degradation of soft tissue. Assuming that tissue is considered irreversibly damaged or necrotic once the damage parameter reaches a value of Ω≥1 [31], the necrotic volume $\theta_{V}\left(t\right)$ can be computed by integrating the spatial domain in which this condition is satisfied. In addition to the illustration of the damage parameter, Ω, and the necrotic tissue volume, $\theta_{V}$, in a schematic representation of the setup (see Figure 1), a more detailed description of the implementation with the used parameter values can be found in [16].

Additionally, the maximum tissue temperature over time was used as the second output variable and determined as:

(9) $$T_{\max}\left(t\right)=\max_{r,z}\left\{T\left(r,z,t\right)\right\},\;t\in \left[1,3\right]\;s,$$

where *r* and *z* denote the radial and axial coordinates in the cylindrical domain. The geometric dimensions of the 2-dimensional (2D) axisymmetric FE model, which encompasses both the electrode and tissue, as well as all other boundary and initial conditions, and meshing parameters, were implemented identically to the setup reported in [16]. Therefore, only an outline of the important implementation details is provided here.

The FE model was implemented as a 2D axisymmetric representation, consistent with our previous study. This approach defines a cylindrical tissue domain with a height and radius of 1.5 cm. A ball electrode with a diameter of ⌀4mm was positioned at the center of the top surface. A transition stem with a height and radius of 1mm was attached to the sphere, incorporating a fillet radius of 0.8 mm at the junction between the stem and the ball segment.

At the beginning of the simulation, the electrode and the tissue were in initial contact, with no externally applied forces or internal contact forces between the two bodies. Additionally, no initial displacement fields or structural velocity fields were prescribed. The initial electrode potential in both domains was set to 0V. The initial temperature of the electrode was defined as 19 °C, while the tissue temperature was set to 21 °C.

From a mechanical perspective, the lower boundary of the tissue block was defined as a roller boundary condition, thereby constraining structural displacement in the direction normal to the surface while allowing tangential movement. All remaining external boundaries were free to deform. Additionally, the ball electrode surface was defined such that it could come into contact with the upper tissue surface. The fully coupled augmented Lagrangian contact method was used to solve this contact problem. As in [16], contact was assumed to be frictionless.

From a thermal perspective, the lower boundary of the tissue was maintained at a constant temperature of 21 °C. The upper surface of the electrode stem was defined as thermally insulated, thereby preventing any heat flux across this boundary. All other exposed surfaces of both the tissue and the electrode (provided they are not in mutual contact) were subject to convective heat transfer to the surrounding environment. A convective heat transfer coefficient of $25\;W/(m^{2}K)$ and an ambient temperature of 21 °C were assumed.

From an electrical standpoint, the lower surface of the tissue was defined as the neutral electrode with an electric potential of $V_{\mathrm{NE}}=0\;V$, while the upper surface of the electrode stem was assigned to an RMS potential of $V_{\mathrm{rms}}=98\;V$. This electrical potential was applied only for t≥1s. All remaining outer surfaces of the tissue and the electrode that were not in contact were treated as electrically insulated.

To address the research question, the implemented FE model was solved using a time-dependent study of three seconds. A schematic representation of the simulation procedure is provided in Figure 1. The first second of the simulation involves the displacement of the electrode towards the tissue, to establish contact, to create the interface $\Gamma_{\mathrm{et}}$. The displacement is applied through the displacement vector u, following the governing equation of motion. From the beginning of the 2nd. second to the end of the 3rd. second, a constant electrical voltage is applied to the electrode. This results in Joule heating of the tissue and the subsequent thermally induced tissue necrosis.

It is important to emphasize that the electrode–tissue contact area remains constant after contact is established and thus from the first second until the end of the simulation. This implies that the modeling does not account for tissue shrinkage due to thermally induced protein denaturation and the evaporation of tissue water. This constraint is required to decouple the geometric contact dynamics (e.g., shrinkage or contraction caused by desiccation and protein denaturation) from the parameter sensitivity analysis.

The model’s triangular mesh was generated using the COMSOL mesh generator, with manual control of the element size and growth rate to accurately resolve the strongly nonlinear electro-thermal behaviors. To ensure sufficient resolution of steep gradients in the electrode–tissue contact region, the upper boundary of the tissue domain was subdivided into a refined zone extending 2 mm radially from the symmetry axis. The maximum element size was set to 1.29 mm in the electrode and 0.75 mm in the bulk tissue, while the electrode–tissue interface was locally refined to a maximum element size of 25μm. The element growth rate within the tissue domain was limited to 1.03 to avoid abrupt mesh transitions. The final mesh consisted of 18,344 triangular elements. All simulations were performed using the fully coupled, linear direct solver PARDISO, as implemented in COMSOL Multiphysics.

### 2.2. Parameters Sensitivity Analysis

To assess the impact of parameter variability on the dynamics of maximum temperature and necrosis volume development during electrosurgical monopolar soft coagulation, the selected tissue properties for the sensitivity analysis should comprehensively represent realistic variations within biologically plausible limits for soft tissue. Therefore, the FDA Guideline Premarket Notification (510(k)) Submissions for Electrosurgical Devices for General Surgery was used as a supporting basis, as our modeled application falls under a general electrosurgical procedure. The guideline recommends liver, kidney, and muscle tissue in order to have a good range of variability for general soft tissue indications [33]. The IT’IS database for thermal and electromagnetic parameters of biological tissues [34] was used to compare those three soft tissue properties to find reasonable values for our study. The parameters relevant to this analysis are the temperature-dependent tissue parameters, electrical conductivity, thermal conductivity, and effective heat capacity.

The electrical conductivity across the three tissue types reveals significant variability. For instance, starting from liver tissue with a conductivity of 0.13S/m at a frequency of 350 kHz, kidney tissue exhibits a conductivity of 0.21S/m, which is already 61.5% higher. Muscle tissue further increases this value by 99% to 0.418S/m. For the thermal conductivity, the variation between the tissue types is less pronounced. Kidney has a reported conductivity of 0.53W/(m·K), liver 0.52W/(m·K), while muscle tissue is even lower at 0.49W/(m·K). We used a thermal conductivity of 0.5W/(m·K) and varied it symmetrically by ±6%, corresponding to a range of ±0.03W/(m·K).

The difference in effective heat capacity across the three tissue types is also relatively small, with muscle tissue standing out. Kidney tissue exhibits a heat capacity of 3763J/(kg·K), liver 3540J/(kg·K), and muscle tissue 3421J/(kg·K). For the study, liver tissue’s effective heat capacity was varied symmetrically by ±5%, corresponding to a range of ±177J/(kg·K), encompassing the majority of differences among these tissue types.

To investigate the effect of parameter variability under different electrode–tissue contact areas, 27 tissue parameter configurations were simulated with seven electrode displacement vectors u. Displacement was applied exclusively in the z-direction, incremented from $u_{z}=0.5\;\mathrm{mm}$ to 2mm in 0.25mm steps. Consequently, seven different contact areas $M_{\mathrm{cs}}\left(u\right)$ are established between the electrode and the tissue. A batch sweep of the tissue parameter configurations was combined with an auxiliary sweep on the displacement vector u. All configurations were solved through the implementation of eight parallel computations, with each computation utilizing eight cores of our 64-core AMD Ryzen processor. The total computation time for the simulation was 12 h, 37 min, and 30 s.

A selection of seven configurations was defined from the 27 different tissue parameter configurations in order to investigate variations in the three tissue parameters on the output variables in a detailed analysis. The selected values for each of the three parameters correspond to the reference value at the reference temperature of the modeled parameter σ(T) in (4), k(T) in (5), and $C_{\mathrm{eff}}\left(T\right)$ in (6). The seven configurations represent a variation of each parameter, with the other two coefficients kept constant. These seven configurations are listed in Table 1. All other parameter values used are listed in Table 2.

To assess the sensitivity of the model output with respect to variations in tissue parameters at different contact areas, we employed the normalized local sensitivity measure *S*. This measure uses forward finite differences between adjacent parameter levels. The purpose of this metric is to quantify the extent to which a result ξ(t) changes in relation to variations in a parameter $x_{\mathrm{ref}}$. In this context, we evaluated the time-dependent sensitivity of the two primary model output quantities, the maximum temperature $T_{\max}\left(t\right)$ and the necrotic volume fraction $\theta_{V}\left(t\right)$, with respect to the reference parameter $x_{\mathrm{ref}}$ (with x∈{σ,k,C}) over the active voltage application interval ($t\in \left[1\;s,\;3\;s\right]$). For each reference parameter, the three previously defined tissue parameter values $x_{\mathrm{ref}}^{\left(1\right)}<x_{\mathrm{ref}}^{\left(2\right)}<x_{\mathrm{ref}}^{\left(3\right)}$ were considered. The relative time-dependent sensitivity is thus defined as:

(10) $$S_{x_{\mathrm{ref}}}^{\xi }\left(t\right)=\frac{\left(\xi \left(t;x_{\mathrm{ref}}^{\left(j\right)}\right)-\xi \left(t;x_{\mathrm{ref}}^{\left(i\right)}\right)\right)x_{\mathrm{ref}}^{\left(2\right)}}{\xi \left(t;x_{\mathrm{ref}}^{\left(2\right)}\right)\left(x_{\mathrm{ref}}^{\left(j\right)}-x_{\mathrm{ref}}^{\left(i\right)}\right)},\;\left(i,j\right)\in \left\{\left(1,2\right),\left(2,3\right)\right\}.$$

For the necrotic volume fraction $\theta_{V}\left(t\right)$, the sensitivities were computed only after the onset of necrosis, which is defined as:

(11) $$t_{\mathrm{onset}}=\min\left\{t|\theta_{V}\left(t\right)>0\right\},$$

and thus for $t\geq t_{\mathrm{onset}}$, to avoid a division by zero.

To bring the sensitivity measure to a more general quantity for analyzing it with respect to different contact areas $M_{\mathrm{cs}}$, the time-averaged sensitivity for $\theta_{V}\left(t\right)$ and $T_{\max}\left(t\right)$ was determined. They are defined as:

(12) $${\bar{S}}_{x_{\mathrm{ref}}}^{\theta_{V}}=\frac{1}{t_{2}-t_{\mathrm{onset}}}\int_{t_{\mathrm{onset}}}^{t_{2}}\left|S_{x_{\mathrm{ref}}}^{\theta_{V}}\left(t\right)\right|\;dt,$$

and

(13) $${\bar{S}}_{x_{\mathrm{ref}}}^{T_{\max}}=\frac{1}{t_{2}-t_{1}}\int_{t_{1}}^{t_{2}}\left|S_{x_{\mathrm{ref}}}^{T_{\max}}\left(t\right)\right|\;dt.$$

## 3. Results

**Figure 2:**
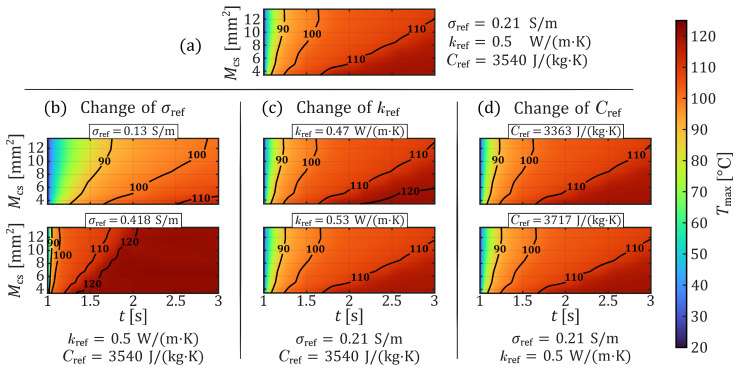
Simulated maximum tissue temperature over time and contact area, for the seven parameter configurations from Table 1. (**a**) $T_{\max}\left(t,M_{\mathrm{cs}}\right)$ of the parameter baseline (configuration 2). (**b**) $T_{\max}\left(t,M_{\mathrm{cs}}\right)$ of two different electrical conductivities (configurations 1 and 3). (**c**) $T_{\max}\left(t,M_{\mathrm{cs}}\right)$ of two different thermal conductivities (configurations 4 and 5). (**d**) $T_{\max}\left(t,M_{\mathrm{cs}}\right)$ of two different effective heat capacities (configurations 6 and 7).

**Figure 3:**
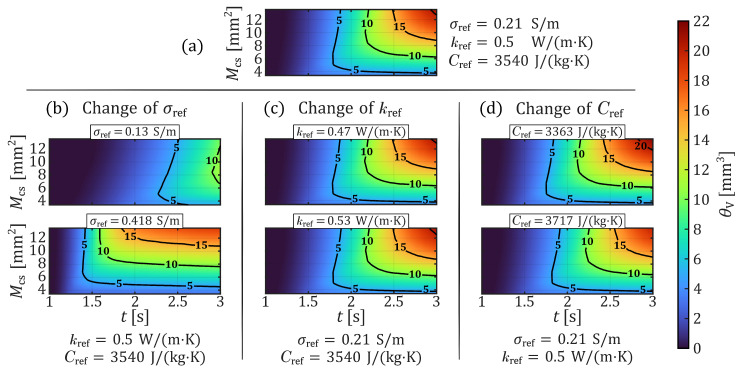
Simulated necrotic tissue volume over time and contact area, for the seven parameter configurations from Table 1. (**a**) $\theta_{V}\left(t,M_{\mathrm{cs}}\right)$ of the parameter baseline (configuration 2). (**b**) $\theta_{V}\left(t,M_{\mathrm{cs}}\right)$ of two different electrical conductivities (configurations 1 and 3). (**c**) $\theta_{V}\left(t,M_{\mathrm{cs}}\right)$ of two different thermal conductivities (configurations 4 and 5). (**d**) $\theta_{V}\left(t,M_{\mathrm{cs}}\right)$ of two different effective heat capacities (configurations 6 and 7).

As shown in Figure 2 and Figure 3 by color gradients, the maximum tissue temperature $T_{\max}$ and the volume of necrotic tissue $\theta_{V}$ at different parameter configurations of $\sigma_{\mathrm{ref}}$, $k_{\mathrm{ref}}$, and $C_{\mathrm{ref}}$ are strongly dependent on the application time *t* and the electrode–tissue contact area $M_{\mathrm{cs}}$. Thus, despite a parameter change, the longer the voltage is applied, the hotter and larger the necrotic area in the tissue will get, and the larger the contact area, the slower the heating process within the tissue. However, the general dependence of the coagulation process on the contact area is not further described in this investigation and was already analyzed in one of our previous studies [16]. Nevertheless, both output quantities, $T_{\max}$ and $\theta_{V}$, clearly highlight the significant influence of the electrical conductivity. This is evident by comparing the plots of $T_{\max}$ in Figure 2a,b as well as of $\theta_{V}$ in Figure 3a,b.

On the other hand, the thermal conductivity and the effective heat capacity exhibit comparatively minor effects on $T_{\max}$ and $\theta_{V}$. As illustrated by the contour plots in Figure 2a,c and Figure 3a,c, an increase in thermal conductivity results in a slightly slower thermal response and marginally smaller necrotic volumes. Similarly, a slight delay in thermal development can be observed in Figure 2a,d as the effective heat capacity increases. However, the differences in the resulting necrotic volumes (Figure 3a,d) appear somewhat more pronounced in this case.

**Figure 4:**
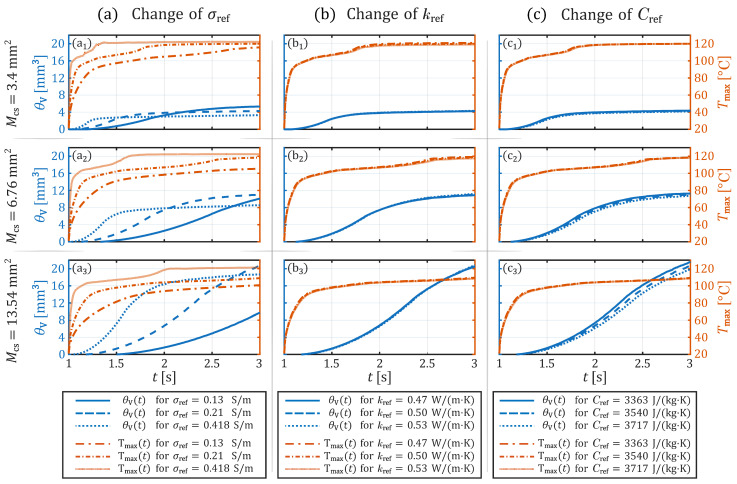
Simulated maximum tissue temperature and necrosis volume over time at different tissue parameters and contact areas. Plot-group (**a**) shows the behavior of $T_{\max}$ and $\theta_{V}$ at the different electrical conductivity values. Plot-group (**b**) illustrates $T_{\max}$ and $\theta_{V}$ at the different thermal conductivity values. Plot-group (**c**) shows the development of $T_{\max}$ and $\theta_{V}$ when the effective heat capacity was varied. Thereby the upper row (graphs **a_1_**–**c_1_**) show the results at a contact area of 3.4 mm^2^, the middle row (**a_2_**–**c_2_**) at 6.76 mm^2^, and the bottom row (**a_3_**–**c_3_**) at 13.54 mm^2^.

Figure 4 shows an alternative visualization of the data from Figure 2 and Figure 3. There, the temporal evolution of the maximum tissue temperature and the necrotic volume is depicted exemplarily for the three contact areas $3.4\;{\mathrm{mm}}^{2}$, $6.67\;{\mathrm{mm}}^{2}$, and $13.54\;{\mathrm{mm}}^{2}$ as line plots. Figure 4a–c illustrates the respective influence of variations in electrical conductivity, thermal conductivity, and effective heat capacity during the 2-s voltage application. The contact area consistently proves to be the most critical parameter affecting the final necrotic volume. Moreover, Figure 4a clearly highlights the significant role of electrical conductivity in influencing the progression of HF current-induced coagulation.

Changes in thermal conductivity exhibit negligible effects on maximum temperature ($\Delta T_{\max}<1\;{}^{\circ}C$) and necrosis evolution ($\Delta \theta_{V}\leq 1.5\%$) (see Figure 4b) variations in effective heat capacity result in minor differences in $\theta_{V}$ at the end of the application period ($t_{2}$) (see Figure 4c). These differences become more pronounced with larger contact areas. Thus, a 5% variation in the effective heat capacity with a contact area of $3.4\;{\mathrm{mm}}^{2}$ results in a necrosis volume difference of 2.4% at the end of the voltage application. The difference in necrosis volume increases further to 2.5% for a contact area of $6.76\;{\mathrm{mm}}^{2}$ (see $c_{2}$ in Figure 4c) and reaches 4% for a contact area of $13.54\;{\mathrm{mm}}^{2}$ (see $c_{3}$ in Figure 4c). However, the temperature profile itself remains largely unaffected (only a difference of ≈0.3 °C at $t_{2}$).

In contrast, changes in electrical conductivity significantly impact both $\theta_{V}\left(t\right)$ and $T_{\max}\left(t\right)$, with larger $M_{\mathrm{cs}}$ amplifying these effects. At the smallest contact area ($M_{\mathrm{cs}}=3.4\;{\mathrm{mm}}^{2}$, in Figure 4a), it can be observed that, at the end of the voltage application, $\theta_{V}$ has reached a plateau phase for the three values of $\sigma_{\mathrm{ref}}$. The saturation of $\theta_{V}$ can be characterized by forming a necrosis plug at the contact site, which prevents Joule heating in deeper tissue regions due to its high-resistance properties. The absolute difference in $\theta_{V}$ at $t_{2}$ between 0.13 S/m and 0.21 S/m is approximately 24.4%, increasing to 29.1% between 0.21 S/m and 0.418 S/m.

For $M_{\mathrm{cs}}=6.76\;{\mathrm{mm}}^{2}$ (see Figure 4a), it is evident that $\theta_{V}$ has not yet reached its saturation phase when $\sigma_{\mathrm{ref}}$ is 0.13S/m at the end of the simulation time. Therefore, when $\sigma_{\mathrm{ref}}$ is 0.13 S/m, $\theta_{V}\left(t_{2}\right)$ is 8.5% smaller compared to $\sigma_{\mathrm{ref}}=0.21\;S/m$. At $\sigma_{\mathrm{ref}}=0.418\;S/m$, $\theta_{V}$ reaches its plateau phase after approximately 0.7 s of voltage activation, after which it continues to increase slowly. However, $\theta_{V}\left(t_{2}\right)$ at 0.418 S/m remains 22.7% smaller than at 0.21 S/m. For $M_{\mathrm{cs}}=13.54\;{\mathrm{mm}}^{2}$ (see Figure 4a), it was determined that only the necrotic volume corresponding to $\sigma_{\mathrm{ref}}=0.418\;S/m$ is in its saturation phase. For the other two values of $\sigma_{\mathrm{ref}}$, the duration of the application time was too short to reach saturation behavior. Consequently, $\theta_{V}\left(t_{2}\right)$ at 0.13 S/m is 52.9% smaller than at 0.21 S/m, while it decreases by 9.9% from 0.21 S/m to 0.418 S/m.

**Figure 5:**
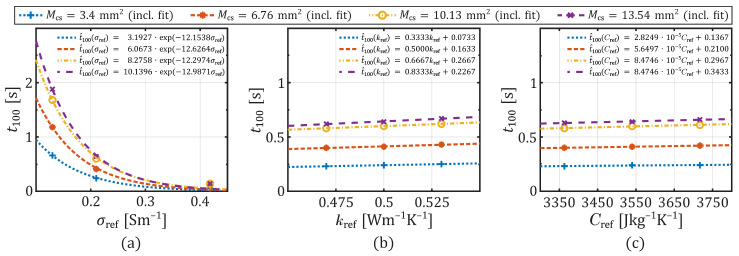
Time to reached the critical temperature threshold of 100 °C depending on tissue properties and contact area sizes: (**a**) $\sigma_{\mathrm{ref}}$, (**b**) $k_{\mathrm{ref}}$, (**c**) $C_{\mathrm{ref}}$ each at four contact areas $M_{\mathrm{cs}}$.

The effect of a change in the reference value of temperature-dependent tissue parameters at four different contact areas on the heating dynamics is shown in the curves of Figure 5a–c. Therefore, the parameter $t_{100}$ serves as a metric to indicate the time point at which the critical temperature of 100 °C was reached within the tissue. The data, together with the trend curves, reveal that increasing the reference in electrical conductivity (see Figure 5a) exponentially accelerates the heating process, i.e., reduces the time to $t_{100}$. Conversely, increasing the reference thermal conductivity (see Figure 5b) or the reference of the effective heat capacity (see Figure 5c) slows the heating process, which is characterized by a positive slope of the trend lines leading to a linear increase in $t_{100}$.

All three graphs further demonstrate a correlation between an increase in the contact area and a corresponding slowdown in the heating process. It appears in Figure 5a that the exponential acceleration of the heating process is amplified through larger contact areas resulting from slower heating at lower conductivity values. Whereas an increase in the reference thermal conductivity or heat capacity results in a slightly slower heating, the larger the contact area is. Thus, as illustrated by the tend curves’ slopes, a change in a tissue parameter exerts a different effect on the heating process for different contact areas.

Furthermore, the curves in the graphs of Figure 5 exhibit saturation behavior with increasing contact area. Consequently, there is a threshold beyond which further enlargement of the contact area has less impact on the heating process and the delay of $t_{100}$ compared to changes in tissue properties. The diminishing distance between the curves towards larger contact areas demonstrates this effect. Additionally, all three graphs show an increase in the slope of the curves with larger contact areas. This indicates a correlation between the contact area and its influence on tissue property variations affecting the heating process.

**Figure 6:**
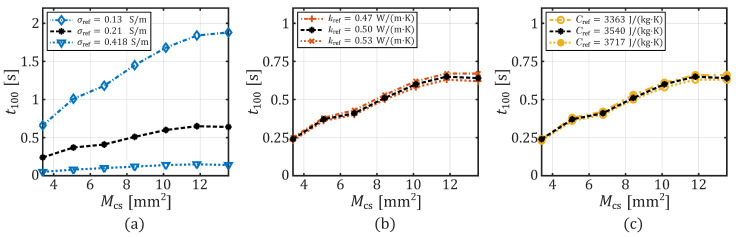
Simulated time to reached 100 °C versus the contact area for different tissue parameters: (**a**) $\sigma_{\mathrm{ref}}$, (**b**) $k_{\mathrm{ref}}$, and (**c**) $C_{\mathrm{ref}}$, with their three variations. The black stars with dashed lines in each graph show the same data resulting from configuration 2 in Table 1.

Figure 6 further illustrates these findings. The graphs demonstrate the extent to which the heating process, measured using the metric $t_{100}$, is influenced by $M_{\mathrm{cs}}$ across the use of different tissue properties. The data, indicated by black stars in each of the three subplots, denote the data of the same parameter configuration and can be seen as a reference. The data displayed has undergone linear interpolation. For the seven tissue property combinations, $t_{100}$ exhibits a saturation behavior as $M_{\mathrm{cs}}$ becomes larger. Additionally, electrical conductivity considerably influences the effect of the contact area on temperature development. For 0.418 S/m (blue triangles in Figure 6a), the absolute change in $t_{100}$ between $M_{\mathrm{cs}}=3.4\;{\mathrm{mm}}^{2}$ and $M_{\mathrm{cs}}=13.54\;{\mathrm{mm}}^{2}$ is only 0.09s. However, this corresponds to a relative change of 180% in $t_{100}$. At an $\sigma_{\mathrm{ref}}$ of 0.21S/m, the absolute change is 0.4s (black stars in Figure 6a), which corresponds to a relative change of 166.6%. The absolute change in $t_{100}$ for $\sigma_{\mathrm{ref}}=0.13\;S/m$ (blue diamonds in Figure 6a) is even 1.22s, which corresponds to a delay in the heating process of 184.8%.

**Figure 7:**
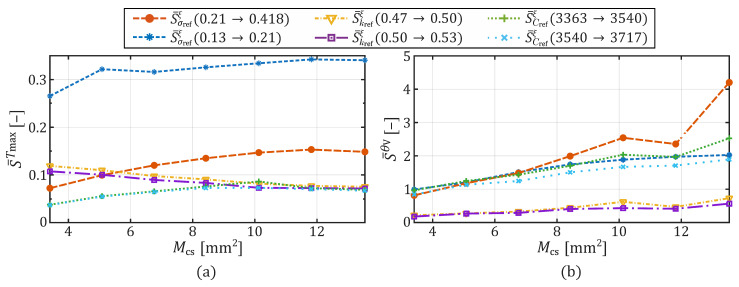
Time-averaged relative sensitivity of $T_{\max}\left(t\right)$ and $\theta_{V}\left(t\right)$ for varied tissue parameter values over an increasing contact area $M_{\mathrm{cs}}$. (**a**) Influence on the thermal peak formation $T_{\max}\left(t\right)$, and (**b**) influence on the tissue necrosis volume development $\theta_{V}\left(t\right)$, each for variations in $\sigma_{\mathrm{ref}}$, $k_{\mathrm{ref}}$, and $C_{\mathrm{ref}}$ across different contact areas $M_{\mathrm{cs}}$.

Figure 7 presents the time-averaged relative sensitivity, after (13) and (12), of $T_{\max}\left(t\right)$ (Figure 7a) and $\theta_{V}\left(t\right)$ (Figure 7b) resulting from variations in the values of $\sigma_{\mathrm{ref}}$, $k_{\mathrm{ref}}$, and $C_{\mathrm{ref}}$, across different contact areas $M_{\mathrm{cs}}$. As illustrated in both graphs, the time-averaged sensitivity varies depending on the contact area for all three parameters. Furthermore, it becomes apparent that the influence of a parameter change across all contact areas on the output quantity $T_{\max}$ is less sensitive than on the output quantity $\theta_{V}$. Thus, the values for ${\bar{S}}^{T_{\max}}$ range between 0.04 and 0.34, whereas ${\bar{S}}^{\theta_{V}}$ ranges between 0.18 and 4.2.

The sensitivity curves in Figure 7a show that for both $k_{\mathrm{ref}}$ and $C_{\mathrm{ref}}$, the time-averaged relative sensitivities obtained for variations from (0.47→0.5) and (0.5→0.53), as well as from (3363→3540) and (3540→3717), are of comparable magnitude and direction, indicating symmetric parameter behavior around the reference value. While the sensitivities for thermal conductivity and heat capacity are similar in terms of direction and magnitude between the upper and lower parameter change ranges, the sensitivities for the upper and lower changes in electrical conductivity are not. When $\sigma_{\mathrm{ref}}$ is changed, it becomes clear that although the curves ${\bar{S}}_{\sigma_{\mathrm{ref}}}^{T_{\max}}\left(0.13\to 0.21\right)$ and ${\bar{S}}_{\sigma_{\mathrm{ref}}}^{T_{\max}}\left(0.21\to 0.418\right)$ are comparable in terms of progression, they differ in magnitude which indicating an asymmetric sensitivity. Thus, ${\bar{S}}_{\sigma_{\mathrm{ref}}}^{T_{\max}}\left(0.13\to 0.21\right)$ moves between 0.27 and 0.34, and ${\bar{S}}_{\sigma_{\mathrm{ref}}}^{T_{\max}}\left(0.21\to 0.418\right)$ between 0.07 and 0.15.

In contrast, the time-averaged relative sensitivity associated with the necrotic volume fraction, ${\bar{S}}^{\theta_{V}}$, depicted in Figure 7b, reveals substantially greater sensitivity to parameter changes and a considerable dependency on the contact area. Specifically, deviations in $\sigma_{\mathrm{ref}}$ and $C_{\mathrm{ref}}$ result in relative sensitivities ranging from 0.99 to 2.03 for ${\bar{S}}_{\sigma_{\mathrm{ref}}}^{\theta_{V}}\left(0.13\to 0.21\right)$, 0.82 to 4.20 for ${\bar{S}}_{\sigma_{\mathrm{ref}}}^{\theta_{V}}\left(0.21\to 0.418\right)$, 0.97 to 2.53 for ${\bar{S}}_{C_{\mathrm{ref}}}^{\theta_{V}}\left(3363\to 3540\right)$, and 0.84 to 1.89 for ${\bar{S}}_{C_{\mathrm{ref}}}^{\theta_{V}}\left(3540\to 3717\right)$ depending on the contact area. Again, an asymmetry to electrical conductivity can be observed, which increases as the contact area increases. Variations in $k_{\mathrm{ref}}$ produce comparatively minor deviations ranging from 0.2 to 0.73 for ${\bar{S}}_{k_{\mathrm{ref}}}^{\theta_{V}}\left(0.47\to 0.5\right)$ and 0.18 to 0.57 for ${\bar{S}}_{k_{\mathrm{ref}}}^{\theta_{V}}\left(0.5\to 0.53\right)$.

## 4. Discussion

This study aims to analyze the effects of electrode–tissue contact area under varying tissue parameters on thermal dynamics and necrosis formation during HF current-induced monopolar coagulation using a multiphysics simulation model. The results highlight the complex interplay between the electrical, thermal, and contact-driven coupled effects on the heating dynamics and necrotic tissue volume. Nevertheless, it is important to note at this point that the observed parameter sensitivities do not represent purely intrinsic tissue behavior, because the electrical contact conductance at the electrode–tissue interface is affected by the temperature-dependent electrical conductivity of the tissue. Parameter variations simultaneously affect volumetric Joule heating and current redistribution at the interface. Consequently, the presented sensitivities characterize not only the properties of the bulk tissue alone, but rather the response of a coupled electrode–tissue system.

In essence, while an influence is evident, the results indicate the presence of mutual dependencies that necessitate careful consideration, particularly in simulation-based virtual sensing approaches. Therefore, the current discussion will first concentrate on the impact that a variation in temperature-dependent tissue parameters exerts on our model’s output. We will then proceed to elucidate how this behavior is affected by differing electrode–tissue contact areas in order to answer our scientific question.

### 4.1. Sensitivity Analysis of Tissue Parameters

As previously demonstrated in studies on the bioheat transfer equation by Hall et al. [12] and Wang et al. [13] for RF ablation, the investigation by Sebek et al. [14] for microwave ablation, or the work by Karaki et al. [15] for a monopolar application, the electrical conductivity of the tissue always emerges as the most critical factor influencing the outcomes. Similarly, in the present simulation, an increase in the electrical conductivity considerably reduces the time required to reach a temperature of 100 °C. Since an increase in electrical conductivity leads to an elevated electric current flow and simultaneously to an increase in electrical contact conductance at the electrode–tissue interface, there is a higher electric current concentration near the contact zone and thus greater electromagnetic losses within the tissue in the close vicinity of the electrode contact. Consequently, this results in accelerated heating dynamics.

A rapid temperature rise associated with high electrical conductivity inhibits, on the other hand, Joule heating in deeper tissue regions and thus influences necrosis formation. This phenomenon is reflected in the plateau observed in our necrotic volume graphs over application time (see Figure 3b and Figure 4a), indicating reduced necrosis propagation. The plateau characterizes a highly resistive necrosis plug built at the complete electrode–tissue contact site. At temperatures near 100 °C, water vaporization in the tissue leads to dehydration and a noticeable reduction in electrical conductivity [38,39]. These nonlinear vaporization processes are incorporated in σ(T), resulting in an abrupt change in conductivity upon reaching the boiling point of water (≈100 °C). Tissue regions exceeding 100 °C become highly resistive due to water loss, significantly diminishing Joule heating in deeper tissue layers. This creates a high-resistance boundary layer near the electrode–tissue site and reduces the effective electrical coupling at the interface, restricting both deeper Joule heating and further necrotic zone expansion. This behavior is clearly reflected in the results obtained. Thus, for the same contact area and an increased electrical conductivity, the resulting volume of tissue necrosis is reduced (see Figure 4a).

Despite the notable differences between the considered application in our analysis towards the RF or microwave ablation (e.g., application time and applied voltage), our findings of the parameters’ impact are consistent with the findings of [12,13,14]. A change in the thermal conductivity or effective heat capacity shows nearly similar effects on the peak temperature development. Both have only a minor effect on the time to reach the boiling point of water, but both lead to slower heating when increased. Thermal conductivity has a slightly greater influence on the maximum temperature development, since the slope of $t_{100}$ is 0.6667, for example, at a moderate contact area of $10.13\;{\mathrm{mm}}^{2}$ (see Figure 5b). Whereas, the slope for a change in effective heat capacity is only $8.4746\;\times \;10^{-5}$ (see Figure 5c). Relatively speaking, when $k_{\mathrm{ref}}$ changes about 5% $t_{100}$ is extended about 2.7%. While a 5% change in $C_{\mathrm{ref}}$ prolong $t_{100}$ about 2.5%.

In contrast to the findings of Karaki et al. [15] our simulations reveal that a change in thermal conductivity has only a slightly greater impact on the output parameter $T_{\max}\left(t\right)$ than the effective heat capacity, but both have a significantly smaller influence on $T_{\max}$ than the electrical conductivity. One potential root cause for this discrepancy could be that we are looking at the dynamics of the maximum temperature rather than the temperature field, and another could be the difference in the modeled heat capacity. It is also noteworthy that the effective heat capacity in our simulation was modeled according to the implementation of Yang et al. [28] and Chen et al. [29], in which the peak of the heat capacity near the boiling point of water is considerably larger than in the model of Karaki et al. [15]. We would therefore expect the influence to be more pronounced in our simulation. However, since the observed phenomenon occurs in the opposite direction, the exact rationale for the discrepancy remains indeterminate at this point.

When analyzing the influence of the parameters on the second output parameter, the thermal necrosis volume (see Figure 3a–d), deviating behaviors are observed. The electrical conductivity still has the largest impact on the output, but the change in effective heat capacity has a greater effect on the necrosis formation than the thermal conductivity. Thus, the time until a necrosis volume of $5\;{\mathrm{mm}}^{3}$ is reached at a contact area of $10.13\;{\mathrm{mm}}^{2}$, for example, shifts by less than 1% with a 5% change in thermal conductivity, whereas the effective heat capacity causes a 2.1% change. These effects can be explained through our governing Equation (1). A change in thermal conductivity affects heat diffusion and smooths the temperature gradient, but has less effect on the thermal energy in the tissue volume. Additionally, it can be assumed that the changes in relation to the short application time are too small for greater effects on the necrosis volume. Therefore, the necrotic formation is less impacted by a change in thermal conductivity.

Conversely, a change in the effective heat capacity exerts a direct and dominant influence on necrosis formation by regulating both the rate of temperature increase and, more importantly, the duration of thermal exposure at critical temperatures. A change of the effective heat capacity alters the local thermal energy storage and, thus, the time-temperature history governing irreversible tissue damage. This finding suggests that an increase in effective heat capacity leads to a reduced temperature rise rate and a prolonged energy accumulation phase, consequently resulting in a slower and less pronounced necrosis formation. When the effective heat capacity decreases, the temperature increases at a slightly faster rate. Therefore, the critical temperature persists for a greater duration within the tissue area. This leads to an increased necrosis volume at the end of the simulation (see Figure 4c). Given that the heat capacity influences both the temperature development and the thermal exposure time, there is an almost proportional effect on the time required (2.2% by a 5% change of the effective heat capacity at $M_{\mathrm{cs}}=10.13\;{\mathrm{mm}}^{2}$) until a necrosis volume of $5\;{\mathrm{mm}}^{3}$ is reached.

### 4.2. Influence of the Contact Area

The present analysis demonstrates that variations in the electrode–tissue contact area substantially affect maximum temperature and necrotic tissue development with regard to changes in temperature-dependent tissue parameters. While variations in thermal conductivity induce only minor changes in thermal response and necrosis formation across all investigated contact areas, the influence of the effective heat capacity on the necrotic volume formation is clearly discernible. The electrical conductivity emerges once more as the tissue parameter with the most pronounced overall impact. Nevertheless, the sensitivities of all investigated parameters exhibit a perceptible dependence on the contact area. This behavior is consistently observed throughout the results and is quantitatively confirmed by the time-averaged relative sensitivity ${\bar{S}}^{T_{\max}}$ and ${\bar{S}}^{\theta_{V}}$, as summarized in Figure 7a and Figure 7b, respectively.

The contact areas that were investigated represent a parametric span from nearly point-like to extended conformal contact. These contact areas are not intended to reflect a statistical distribution of clinical contact conditions. Rather, they span the transition from highly localized, power-density-driven heating at small contact areas to spatially distributed electro-thermal energy deposition at extended conformal contact conditions. Furthermore, to ensure the identifiability of parameter sensitivities, the contact area remained constant during the voltage application within each simulation. Dynamic variations of the electrode position or contact pressure, which may occur in clinical practice, were deliberately excluded, as time-dependent geometry evolution would introduce path-dependent effects that mask parameter-specific sensitivities.

It is essential to emphasize that the dependence on the contact area is not purely geometric. Increasing the contact area results in an alteration of the relative contribution of interface-mediated current redistribution compared to volumetric heat diffusion. For small contact areas, the heating is dominated by concentrated power density. However, for larger areas, the process becomes increasingly influenced by material-dependent electro-thermal coupling at the interface, thereby amplifying parameter sensitivities.

The applied sensitivity metric reveals that variations in $C_{\mathrm{ref}}$ lead to consistently low sensitivities of the time-dependent maximum temperature. For all investigated contact areas, ${\bar{S}}_{C_{\mathrm{ref}}}^{T_{\max}}$ remain below 0.09 and is therefore strongly under-proportional. Although a slight increase in ${\bar{S}}^{T_{\max}}$ can be observed with increasing contact area, the overall impact remains negligible in the present context, as ${\bar{S}}_{C_{\mathrm{ref}}}^{T_{\max}}\ll 1$. Consequently, a 5% variation in $C_{\mathrm{ref}}$ results in a change of the output quantity $T_{\max}$ by less than 0.45%.

A comparable conclusion can be drawn for variations in $k_{\mathrm{ref}}$. In this case, the time-averaged relative sensitivity slightly decreases with increasing contact area. Thus, the largest influence is observed for the smallest contact area, where ${\bar{S}}_{k_{\mathrm{ref}}}^{T_{\max}}$ reaches a value of 0.11 over the investigated parameter range (0.47 to 0.53W/(m·K)), corresponding to a 0.55% change in $T_{\max}$ for a 5% parameter variation. For contact areas exceeding $M_{\mathrm{cs}}>5.9\;{\mathrm{mm}}^{2}$, ${\bar{S}}_{k_{\mathrm{ref}}}^{T_{\max}}$ falls below 0.1, indicating a pronounced underproportional influence.

Although electrical conductivity exhibits the largest absolute influence on the output quantity $T_{\max}$ in the FEM simulation results, its time-averaged relative sensitivity is only moderately higher than those of thermal conductivity and effective heat capacity. This apparent discrepancy can be attributed to the substantially different parameter variation ranges considered in the analysis. While $k_{\mathrm{ref}}$ and $C_{\mathrm{ref}}$ were varied within comparatively narrow intervals (±6% and ±5%, respectively), $\sigma_{\mathrm{ref}}$ was varied over a much broader range (−61.5% to +99%), reflecting the considerable physiological variability between different biological tissue types.

Interindividual variability due to age, physiological condition, or pathological alterations can shift tissue properties within or beyond the investigated parameter ranges. In the context of the presented analysis, variability falling within the investigated ranges corresponds to different parameter sets inside the explored parameter space, whereas parameter values outside this range may lead to quantitatively different sensitivity magnitudes. However, it is important to note that quantitative sensitivity magnitudes may need to be reassessed for tissue types or parameter values that fall outside the covered parameter range.

As the time-averaged relative sensitivity is normalized by the relative parameter variation, large absolute temperature changes induced by variations in $\sigma_{\mathrm{ref}}$ are partly compensated by the corresponding normalization factor. In addition, electrical conductivity predominantly governs the heating dynamics, which are subsequently averaged over the application time when computing ${\bar{S}}^{T_{\max}}$. As a result, the sensitivity metric characterizes the local, normalized system response rather than the absolute effects observed in the simulation results shown in Figure 2, Figure 4, Figure 5 and Figure 6. Moreover, nonlinearities arising from the coupled thermo-mechanical and electro-thermal problem formulation may further reduce the local slope around the reference value, particularly for large parameter variations, thereby reducing contrast in the time-averaged relative sensitivity measures.

The nonlinear behavior observed for variations in $\sigma_{\mathrm{ref}}$ with respect to the characteristic time $t_{100}$, describing the dynamic development of the maximum temperature up to the critical threshold of 100 °C (see Figure 5a), is consistently reflected in the corresponding time-averaged relative sensitivity curves shown in Figure 7a. In particular, the sensitivity associated with a change from 0.13 to 0.21S/m exceeds that obtained for a change from 0.21 to 0.418S/m. The dependence on the electrode–tissue contact area is evident when comparing Figure 6a and Figure 7a, demonstrating that the influence on the temperature dynamics increases with increasing contact area. Despite differences in magnitude, the overall trends of the sensitivity curves ${\bar{S}}_{\sigma_{\mathrm{ref}}}^{T_{\max}}\left(0.13\to 0.21\right)$ and ${\bar{S}}_{\sigma_{\mathrm{ref}}}^{T_{\max}}\left(0.21\to 0.418\right)$ are similar, which is further supported by the relative changes in $t_{100}$ between the smallest and largest contact area.

Overall, the time-averaged relative sensitivities of $T_{\max}$ with respect to $\sigma_{\mathrm{ref}}$, $k_{\mathrm{ref}}$, and $C_{\mathrm{ref}}$ are under-proportional, indicating that normalized local parameter variations lead to less-than-proportional changes in the thermal response. However, this observation must be interpreted in conjunction with the physiological variability of the respective tissue parameters. Electrical conductivity exhibits a substantially broader range across biological tissue types than thermal conductivity and effective heat capacity. Consequently, even an under-proportional normalized sensitivity can result in pronounced absolute temperature variations when realistic inter-tissue variability is considered. Electrical conductivity, therefore, remains a key parameter in electrosurgical monopolar soft coagulation, as its large physiological variation outweighs the reduced normalized sensitivity. This highlights the importance of considering both relative sensitivity measures and the absolute parameter ranges when evaluating the relevance of parameters in bio-electrical-thermal simulations.

When analyzing the time-averaged relative sensitivity of the necrotic tissue volume, a more pronounced difference between $k_{\mathrm{ref}}$ and $C_{\mathrm{ref}}$ becomes apparent. In agreement with the trends observed in the absolute results, Figure 7b shows that increasing the contact area leads to a substantial rise in ${\bar{S}}^{\theta_{V}}$, particularly for variations in $C_{\mathrm{ref}}$. Averaged over the entire parameter range of $C_{\mathrm{ref}}$ (3363 to 3717J/(kg·K)), ${\bar{S}}^{\theta_{V}}$ increases from 0.9 at the smallest contact area, corresponding to a 4.5% change in necrotic volume for a 5% parameter variation, to 2.21 at the largest contact area, yielding an 11.05% change. Thus, for contact areas exceeding approximately $4\;{\mathrm{mm}}^{2}$, variations in $C_{\mathrm{ref}}$ result in a disproportionate response of the necrotic tissue volume (${\bar{S}}_{C_{\mathrm{ref}}}^{\theta_{V}}>1$), highlighting the increasing relevance of effective heat capacity for necrosis formation at larger contact areas.

For $k_{\mathrm{ref}}$, the sensitivity ${\bar{S}}_{k_{\mathrm{ref}}}^{\theta_{V}}$ remains under-proportional across all investigated contact areas. Nevertheless, the average sensitivity increases from 0.2 at the smallest contact area to 0.65 at the largest contact area. Accordingly, a 5% variation in $k_{\mathrm{ref}}$ leads to a change in necrotic volume of approximately 1% and 3.25% for the smallest and largest contact areas, respectively. Although this influence is clearly weaker than that of $\sigma_{\mathrm{ref}}$ and $C_{\mathrm{ref}}$, thermal conductivity should not be entirely neglected. However, it remains the least influential parameter with respect to necrotic tissue volume formation in this study.

The sensitivity of electrical conductivity exhibits an even more pronounced dependence on contact area when considering necrotic tissue volume, as shown in Figure 7b. Averaged ${\bar{S}}^{\theta_{V}}$ values range from approximately 0.8 to 3.6 across the investigated contact areas for $\sigma_{\mathrm{ref}}$ values between 0.13 and 0.418S/m. An asymmetric behavior is again observed between the sensitivity ranges ${\bar{S}}_{\sigma_{\mathrm{ref}}}^{\theta_{V}}\left(0.13\to 0.21\right)$ and ${\bar{S}}_{\sigma_{\mathrm{ref}}}^{\theta_{V}}\left(0.21\to 0.418\right)$. The latter exhibited a stronger increase with contact area, reaching a value of 4.2 at the largest contact area, whereas the former reached only 2.03. This observation indicates that the chosen averaging interval of the parameter variation can substantially influence the interpreted sensitivity values.

As discussed in our previous study [16], larger contact areas lead to more pronounced temperature gradients at the contact edges. This behavior can be attributed to locally concentrated electric fields and comparatively slower heating dynamics in these regions. For smaller contact areas, variations toward higher electrical conductivity result in less pronounced spatial inhomogeneities due to the more rapid and concentrated heating process, thereby reducing sensitivity with respect to necrotic volume. A similar interpretation applies to variations in the effective heat capacity. At larger contact areas, thermal energy is distributed over a greater tissue volume, whereas at smaller areas it remains highly concentrated. Consequently, variations in $\sigma_{\mathrm{ref}}$ and $C_{\mathrm{ref}}$ have less influence on the temperature distribution and necrosis formation at small contact areas, where high energy density dominates tissue heating and destruction. As the contact area increases, this dominance reduces, making the process more sensitive to underlying tissue properties.

Regarding the research question raised in this study, the results obtained clearly demonstrate that the electrode–tissue contact area exerts a substantial influence on the sensitivity of temperature-dependent parameters in electrosurgical monopolar soft coagulation. The maximum temperature development and the extent of tissue necrosis show a pronounced and systematic dependence on the contact area, with electrical conductivity and effective heat capacity being particularly affected. Increasing contact area has been observed to amplify both absolute thermal and necrotic effects. Furthermore, these effects have been found to result in changes to the normalized sensitivity characteristics, particularly with regard to necrotic volume formation. These findings confirm that the electrode–tissue contact area acts as a critical modulating factor in bio-electro-thermal responses of the tissue and must therefore be explicitly considered when evaluating tissue parameter relevance in simulation-based analyses of electrosurgical monopolar coagulation processes.

Since the mathematical model employed in this analysis is identical to the one used in our previous work [16], the reader is referred to that publication for a detailed discussion of the model-specific limitations. Nevertheless, it is important to explicitly outline certain limitations relevant to this sensitivity analysis, which appear to be particularly relevant concerning real electro-thermal and thermo-mechanical effects. The electrode–tissue contact area and thus the contact geometry were assumed to remain constant throughout each simulation. But in clinical applications, dehydration and protein denaturation, which also lead to tissue shrinkage, as well as electrode displacements due to user handling, may alter the effective contact area and local pressure distribution. These electrode–mechanical feedback mechanisms are not captured in the present model and may influence absolute temperature values and necrosis volumes. However, inclusion of dynamic contact evolution would prevent isolation of individual parameter sensitivities on the contact area itself, which is the primary objective of this analysis.

In the context of the present study, it is important to note that the model has not yet been experimentally calibrated to reproduce absolute in vivo or ex vivo temperature or necrosis volume values. Consequently, the reported maximum temperatures and necrotic volumes should be interpreted as relative quantities within the defined multiphysical model framework rather than as direct predictions for specific clinical scenarios. The conclusions of this work are therefore based on the identified parameter trends and their interdependencies under controlled boundary conditions. Since the governing equations and constitutive relations are derived from established physical formulations, the simulations allow consistent analysis of how parameter variations propagate through the coupled electro-thermal system. However, quantitative agreement with experimental measurements would require dedicated calibration and validation studies beyond the scope of the present investigation. Therefore, the reported quantitative differences do not represent statistically derived variability but relative sensitivity trends within the defined model configuration and parameter space. These trends serve as indicators of the structural response characteristics of the coupled electro-thermal system under controlled boundary conditions.

Overall, this investigation demonstrates that variations in the temperature-dependent tissue parameters influence heating dynamics and necrosis tissue formation in a distinctly contact-area-dependent manner. The results reveal that the electrode–tissue interface governs how electro-thermal interactions evolve within the coupled system, thereby modulating the relative importance of individual tissue parameters. Among the investigated parameters, electrical conductivity consistently emerges as the dominant factor across all contact conditions, highlighting the importance of accurately characterizing tissue electrical properties during HF surgical procedures.

At the same time, the findings indicate that reliable knowledge or estimation of the electrode–tissue contact area is essential, as identical energy delivery settings may lead to fundamentally different heating outcomes depending on the interface condition. From a control perspective, this suggests that future generators or control strategies may benefit from incorporating contact-state information when defining voltage or power delivery. Adaptive operating concepts that account for electrode–tissue interface conditions could therefore improve the predictability and robustness of coagulation outcomes. In this context, the identified sensitivity relationships provide a causal mechanism basis for the development of contact-aware control strategies and physics-based virtual sensing approaches.

Regarding parameter relevance, effective heat capacity becomes increasingly important at larger contact areas, where it can exert a disproportionate influence on necrotic volume formation, whereas thermal conductivity remains of comparatively minor importance under the investigated monopolar soft coagulation conditions.

Taken together, these findings emphasize that the electrode–tissue contact area constitutes a critical interface parameter that must be explicitly considered in physics-based electro-thermal simulations. Models that consistently capture this coupling provide an important theoretical basis for future virtual sensing concepts, enabling model-based estimation of tissue state and supporting the development of sensor-informed monitoring and control strategies aimed at improving procedural robustness, precision, and patient safety in HF surgical applications.

## 5. Conclusions

In this study, a physics-based FE model was utilized to investigate how the electrode–tissue contact area influences the sensitivity of temperature-dependent tissue parameters in electrosurgical monopolar soft coagulation. By systematically varying electrical conductivity, thermal conductivity, and effective heat capacity across multiple contact areas, the impact of contact conditions on the dynamics of maximum temperature and necrotic tissue volume formation was quantified with a controlled multiphysical framework. The results demonstrate that the electrode–tissue contact area is a key modulating factor governing relative parameter sensitivity. Electrical conductivity was identified as the dominant parameter influencing heating dynamics and necrosis formation across all investigated contact areas, whereas thermal conductivity exhibited only a minor effect. Conversely, the effective heat capacity exhibited a significant contact-area-dependent influence on necrotic volume formation, with disproportionate sensitivity observed at extended contact conditions.

These findings highlight that the electrode–tissue interface must be explicitly represented in physics-based electro-thermal models when assessing parameter influence. Neglecting contact-area effects may distort the interpretation of parameter relevance within such modeling approaches. The identified contact-area-dependent sensitivity shifts indicate that interface conditions can alter parameter dominance within electro-thermal models, which has implications for uncertainty analysis and model-based state estimation. From a methodological perspective, the presented analysis clarifies how parameter variations propagate through a coupled electro-thermal system and provides a structural basis for future developments in model-based state estimation and control concepts in electrosurgical applications.

## Figures and Tables

**Table 1:** Different initial tissue parameter configurations.

Configuration	$\sigma_{\mathrm{ref}}$ [S/m]	$k_{\mathrm{ref}}$ [W/(m·K)]	$C_{\mathrm{ref}}$ [J/(kg·K)]
1	0.13	0.50	3540
2	0.21	0.50	3540
3	0.418	0.50	3540
4	0.21	0.47	3540
5	0.21	0.53	3540
6	0.21	0.50	3363
7	0.21	0.50	3717

**Table 2:** List of the model’s material parameters.

Parameter			Value	Unit	Source
Stainless steel ball electrode					
	Electrical conductivity	$\sigma_{e}$	1.37 × 10^6^	S/m	
	Thermal conductivity	$k_{e}$	15	W/(m·K)	
	Density	$\rho_{e}$	7900	$\mathrm{kg}/m^{3}$	
	Poisson’s ratio	$\nu_{e}$	0.29		[35]
	Young’s modulus	$E_{e}$	200	GPa	[35]
Biological tissue					
	Density	$\rho_{t}$	1079	$\mathrm{kg}/m^{3}$	[34]
	Poisson’s ratio	$\nu_{t}$	0.5		
	Young’s modulus	$E_{t}$	1980	Pa	[36]
	Frequency factor	$A_{0}$	$7.39\times 10^{39}$	$s^{-1}$	[37]
	Activation energy	$E_{a}$	$2.577\times 10^{5}$	J/mol	[37]

## Data Availability

The data supporting the conclusions of this study are contained within the article. Additional data are available from the corresponding author upon reasonable request.

## Abbreviations

The following abbreviations are used in this manuscript:

2D	2-dimentional
AC	Alternating Current
FDA	Food and Drug Administration
FE	Finite Element
FEM	Finite Element Modeling
HF	High-Frequency
PDE	Partial Differential Equation
RF	Radiofrequency
RMS	Root-Mean-Square
